# A genomics-based investigation of acetic acid bacteria across a global fermented food metagenomics dataset

**DOI:** 10.1016/j.isci.2025.112139

**Published:** 2025-03-01

**Authors:** Erkang Zhang, Samuel Breselge, Niccolò Carlino, Nicola Segata, Marcus J. Claesson, Paul D. Cotter

**Affiliations:** 1 Teagasc Food Research Centre, Cork, Ireland; 2 School of Microbiology, University College Cork, Cork, Ireland; 3 APC Microbiome Ireland, Cork, Ireland; 4 Department of Cellular, Computational and Integrative Biology, University of Trento, Trento, Italy

**Keywords:** Microbial genomics, Microbial metabolism, Food microbiology

## Abstract

Developing a better understanding of the genomic and metabolic features of acetic acid bacteria (AABs) has the potential to facilitate an improvement of the taste or flavor of fermented foods. Here, we conducted a high-resolution analysis of AABs present in fermented foods based on the investigation of 337 high-quality metagenomic-assembled genomes (MAGs) recovered from 223 metagenomic samples. Firstly, by integrating these MAGs, we built a phylogenetic tree of high-quality MAGs using GTDB-Tk. We found that AABs MAGs from food-related samples and those from other environments are generally phylogenetically distinct, with the majority of those from fermented foods being assigned to a relatively small number of genera. Functional metagenomic analysis also revealed that the fermented food-associated AABs MAGs are associated with the production of carbohydrate-active enzymes, antibiotic resistance genes, and secondary metabolites. Through these investigations, we have gained substantial insights into the diversity, function, and roles of AABs in fermented food microbiomes.

## Highlights

• A broad MAG database enabled a deeper investigation into the AABs in fermented foods
• Food-related AABs MAGs are phylogenetically distinct from those in other environments
• Nine putative novel AABs species were detected from high-quality MAGs
• Function traits differ between AABs species or fermented food AABs communities

## Graphical abstract

**Figure:**
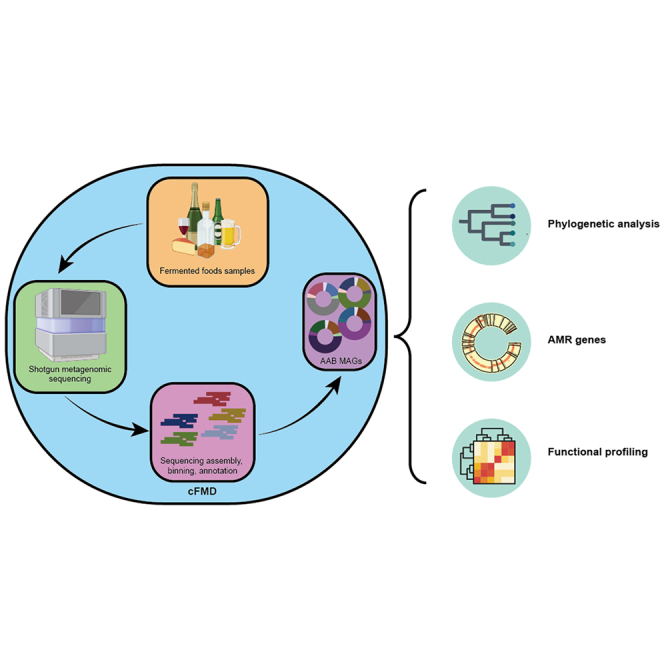

## Introduction

Since the Neolithic age, fermentation has played a role in preserving food.^1^ Food fermentation continues to be used, from homes to large commercial plants, not only to prevent food spoilage but also to produce unique sensory characteristics, such as flavor, smell, and color.^2^ In addition, various fermented foods have been shown to have beneficial effects on human health due to the microbes present, the metabolites produced, or other characteristics.^3^^,^^4^^,^^5^^,^^6^ As a result, levels of consumption of fermented food are increasing across many countries, and there have been increased calls for such foods to be included more widely among national dietary guidelines.^7^^,^^8^ The microorganisms present in fermented foods are selected according to, for example, the raw materials, the manner in which they are stored and the frequently extreme environments, such as high salinity or high acidity, that are present at the beginning or during the course of the fermentation process.^9^^,^^10^ Many of the selected strains associated with fermented foods have an ability to synthesize important bioactive molecules.^11^ Thus, understanding the taxonomy, distribution, and predicted function of the microbial communities in fermented foods may have considerable value with respect to the future further optimization of these foods.

Acetic acid bacteria (AABs) are obligately aerobic bacteria within the family *Acetobacteraceae* and are well known from an applied perspective for their role in the production of acetic acid, vitamin C, or cellulose.^12^^,^^13^ These bacteria also play important roles in the production of a variety of fermented foods, beverages, and condiments, such as kefir, kombucha, and vinegar. However, AABs are not studied to the same extent^14^ compared with other food-grade and industrially important microorganisms in fermented food, such as lactic acid bacteria (LAB).^15^^,^^16^^,^^17^ Thus, although AABs are believed to play an important role in many fermented foods and could be harnessed to improve the quality and/or flavor of fermented products,^18^ it could be argued that their full potential has yet to be harnessed. For other fermented food-associated microorganisms, high-throughput DNA sequencing and bioinformatic analysis has already shown to have the capability of addressing this need.^19^^,^^20^^,^^21^

Indeed, whole-metagenome shotgun sequencing (WMGS) presents an unprecedented opportunity to profile taxonomic composition and functional potential of microbial communities and to recover whole-genome sequences.^22^ The main advantage offered by WMGS is that it can theoretically facilitate the genomic analysis of all microorganisms in the sample, including yet-to-be-cultured microbes. After assembling short metagenomic reads obtained from shotgun sequencing into contiguous segments of DNA, additional bioinformatics tools can be used to complete metagenomic binning, the recovery of metagenomic-assembled genomes (MAGs), and in-depth analysis of these MAGs. To date, a detailed analysis of AABs MAGs derived from fermented foods has yet to be performed.

In this study, a combination of bioinformatics tools were used to characterize 337 high-quality AABs MAGs recovered from 223 fermented food metagenomic samples from within the curatedFoodMetagenomicData (cFMD), including, for example, kombucha, milk kefir, water kefir, and sourdough. The insights provided greatly expand our knowledge of the taxonomy and metabolic potential of AABs in a manner that can further guide their application in utilization by food and other industries.

## Results

### AABs MAGs derived from fermented foods are more homogeneous than those of other origin

**Figure 1:**
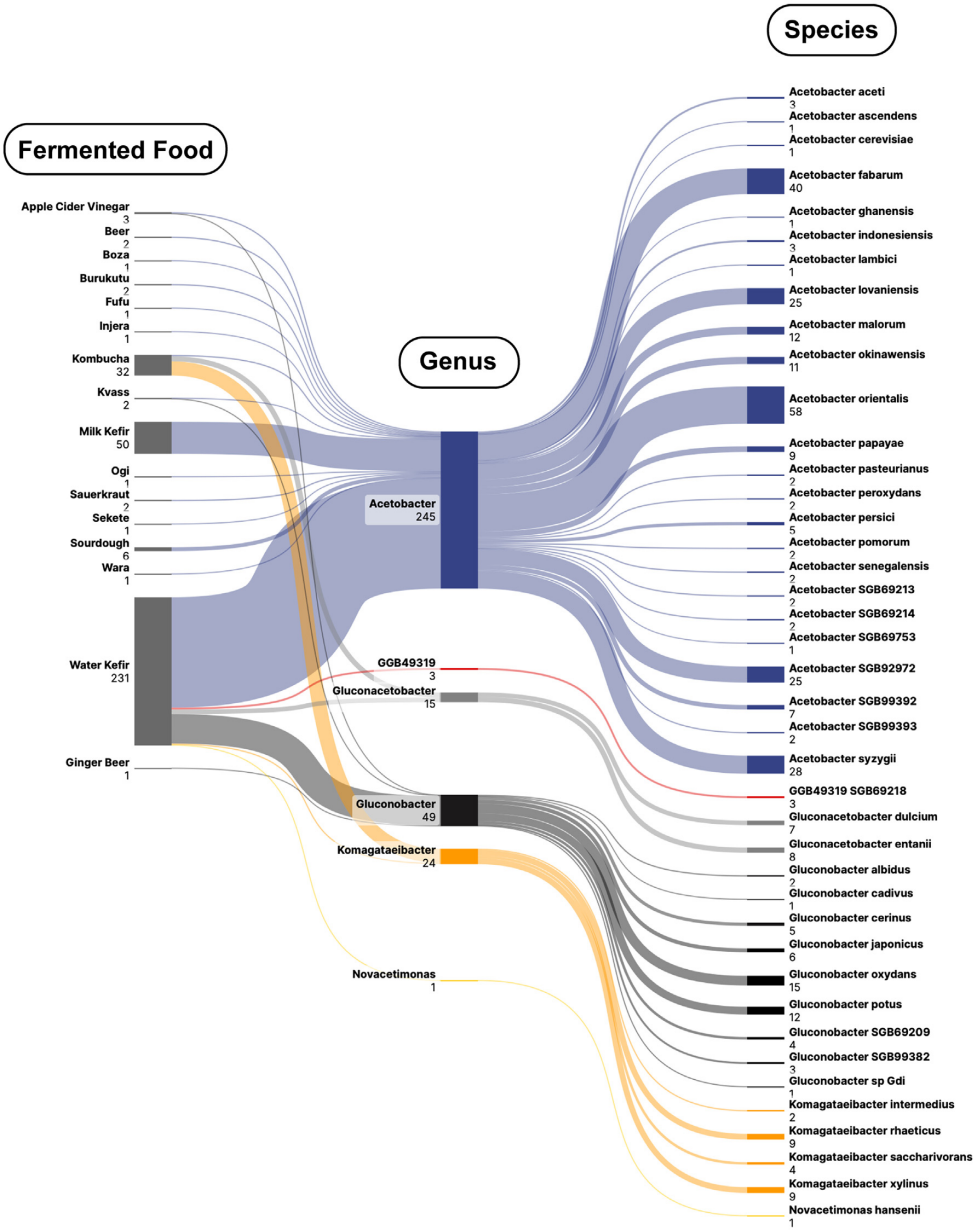
The microbial composition and diversity of AABs in fermented foods Left: fermented foods; middle: genus; right: species. The numbers in the figure represent the quantity of MAGs.

Within the newly created cFMD (https://github.com/SegataLab/cFMD), AABs MAGs were identified in 322 metagenomic food samples. These samples could generally be classified as those that resulted from dairy- (cheese, milk kefir, nunu, koumiss, etc.) or plant-based fermentations (kombucha, sourdough, etc.). All AABs MAGs were members of the family *Acetobacteraceae*, with 6 genera and 41 species being identified (Figure 1). We found strong links between AABs species and food types: all AABs MAGs derived from milk kefir belonged to *Acetobacter* spp., water kefir hosted mainly *Acetobacter* spp. (74.46%) and *Gluconobacter* spp. (19.91%), while *Komagataeibacter* spp. (68.75%) and *Gluconacetobacter* spp. (25%) were most common in kombucha. Nine out of 43 species-level genome bins (SGBs) were annotated as unknown SGBs, potentially representing 9 new species, including 6 *Acetobacter* spp., 2 *Gluconobacter* spp., and an unknown genus-level genome bin (*GGB49319 SGB69218*). There were 3 MAGs assigned to this unknown genus-level genome bin, which all came from water kefir, with the highest identity match (78%) for these 3 MAGs being *Acidisphaera rubrifaciens*.

**Figure 2:**
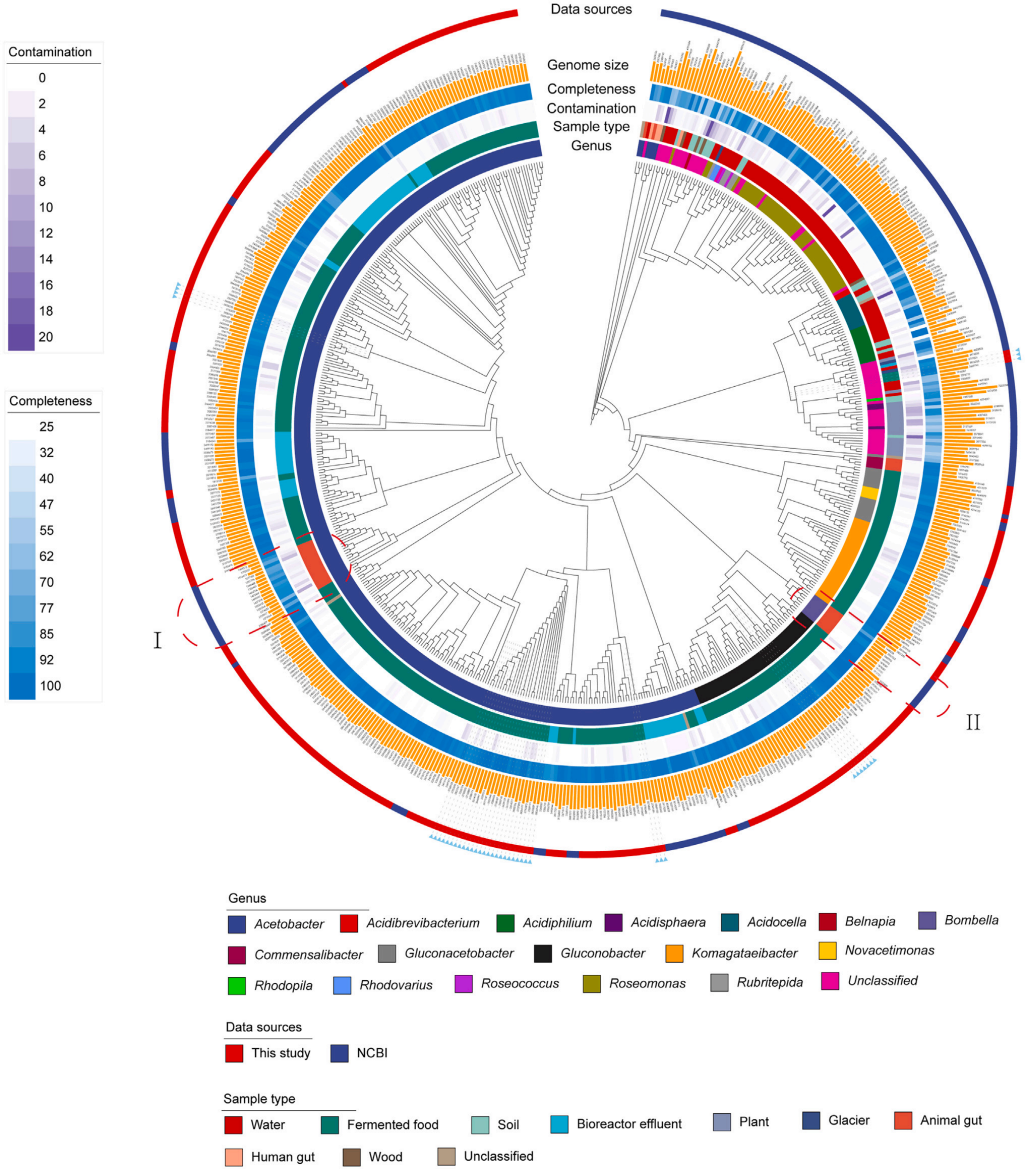
Phylogenetic tree of 609 AABs MAGs (337 high-quality AABs MAGs referred to cFMD and 272 AABs MAGs from other studies), with outer rings showing the metadata for each MAGs The blue arrows indicate which MAGs are potentially novel species.

To gain a deeper insight into the genetic relationships between the various AAB, a phylogenetic analysis was conducted, which included the 337 high-quality AABs MAGs referred to earlier and a further 272 AABs MAGs from other studies (Figure 2). The extended AABs MAG dataset included strains reconstructed from human, animal, food, or environmental samples (such as water, soil, and glaciers). As shown in Figure 2, MAGs clustered primarily according to their origin. The majority of MAGs had a food-related origin, being reconstructed from samples of fermented foods or bioreactor effluent from the food factory and clustered together and distinctly from all others, with the exception of two clades from animal ruminants samples^23^ (I; *Acetobacter* sp.) and honeybees^24^ (II; *Bombella apis*). While most of the AABs derived from fermented foods were assigned to *Acetobacter*, *Gluconobacter*, *Komagataeibacter*, and a small number of other species, other AABs of an environmental origin clustered distinctly, establishing that these environments do not represent a common source of the AABs found in fermented foods.

### Carbohydrate utilization function analysis revealed significant differences among AABs MAGs from milk kefir, water kefir, and kombucha

**Figure 3:**
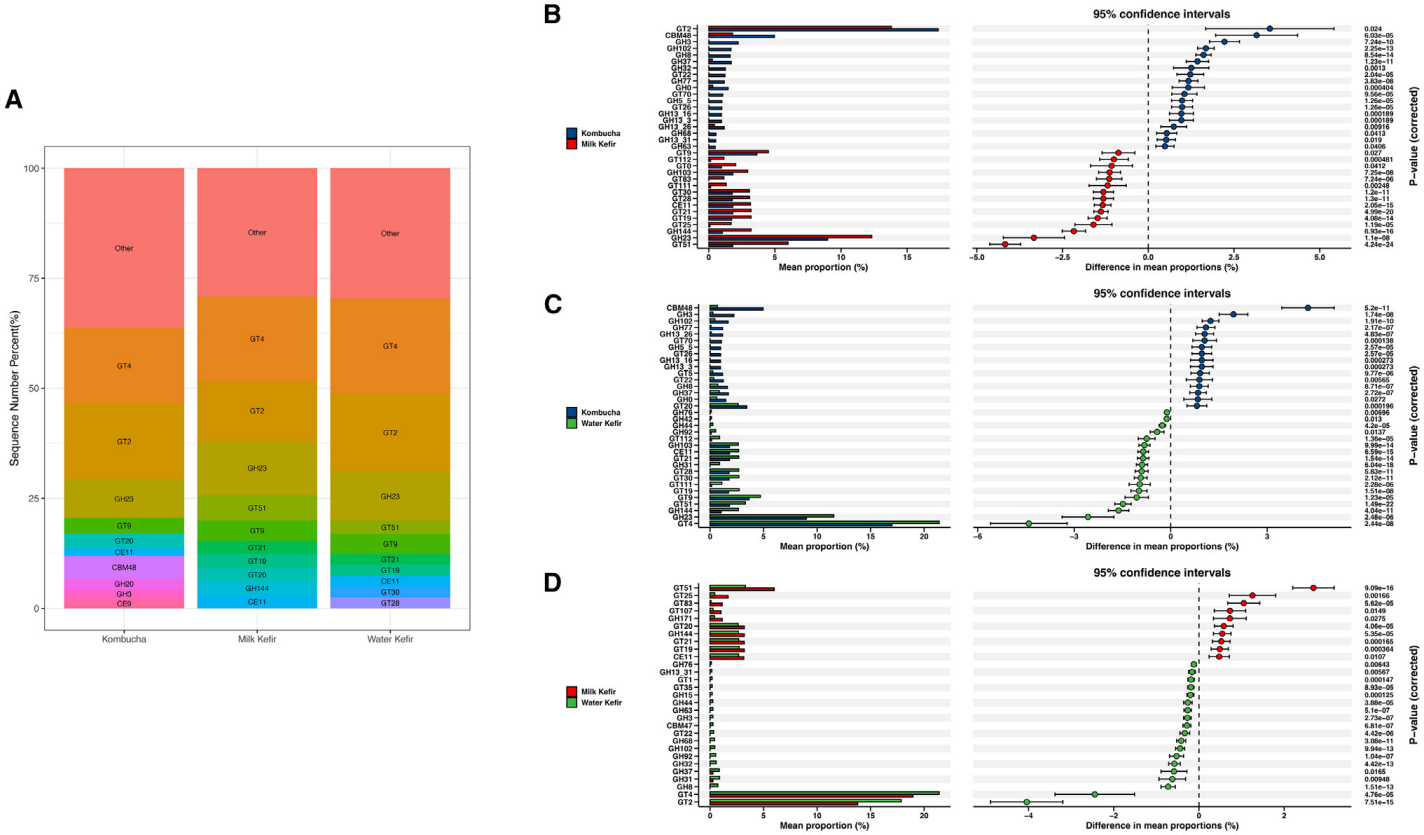
CAZyme annotation of AABs MAGs derived from kombucha, water kefir, and milk kefir (A) Top 10 CAZyme families and their relative abundance in AABs MAGs. (B–D) Pairwise comparison of the metagenome-encoding microbial carbohydrate metabolism genes across the three types of fermented foods. Data are represented as mean ± SEM.

Carbohydrate-active enzymes (CAZymes) are enzymes involved in the synthesis, metabolism, and recognition of complex carbohydrates^25^ and thus can play a key role in the fermentation of food substrates. In addition to identifying the CAZymes encoded by the AABs present in fermented foods, we compared their distribution across the three fermented foods (milk kefir, water kefir, and kombucha) from which the largest number of AABs MAGs was derived. The analysis revealed that the most common CAZymes encoded were glycoside hydrolases (GHs) and glycosyltransferases (GTs) (Figure 3A), which are enzymes associated with the utilization of sugars. Specifically, the relative abundance of GH4 and GT2 genes was more common than that of other CAZyme genes in these fermented foods (Figure 3A). A more specific examination of the ten CAZymes with the highest relative abundance across the three fermented foods revealed differences in the CAZyme family profile for kombucha relative to the other two fermented foods (Figure 3A). Indeed, a pairwise comparison of the metagenome-encoding microbial carbohydrate metabolism genes across the three types of fermented foods (Figures 3B–3D) revealed that a number of CAZyme families (GT2, carbohydrate-binding module 48 [CBM48], and GT3) were significantly enriched in kombucha compared with milk kefir and that the relative abundance of GT4 and GH23 families was significantly higher in water kefir and that GT51 was significantly enriched in milk kefir AAB.

**Figure 4:**
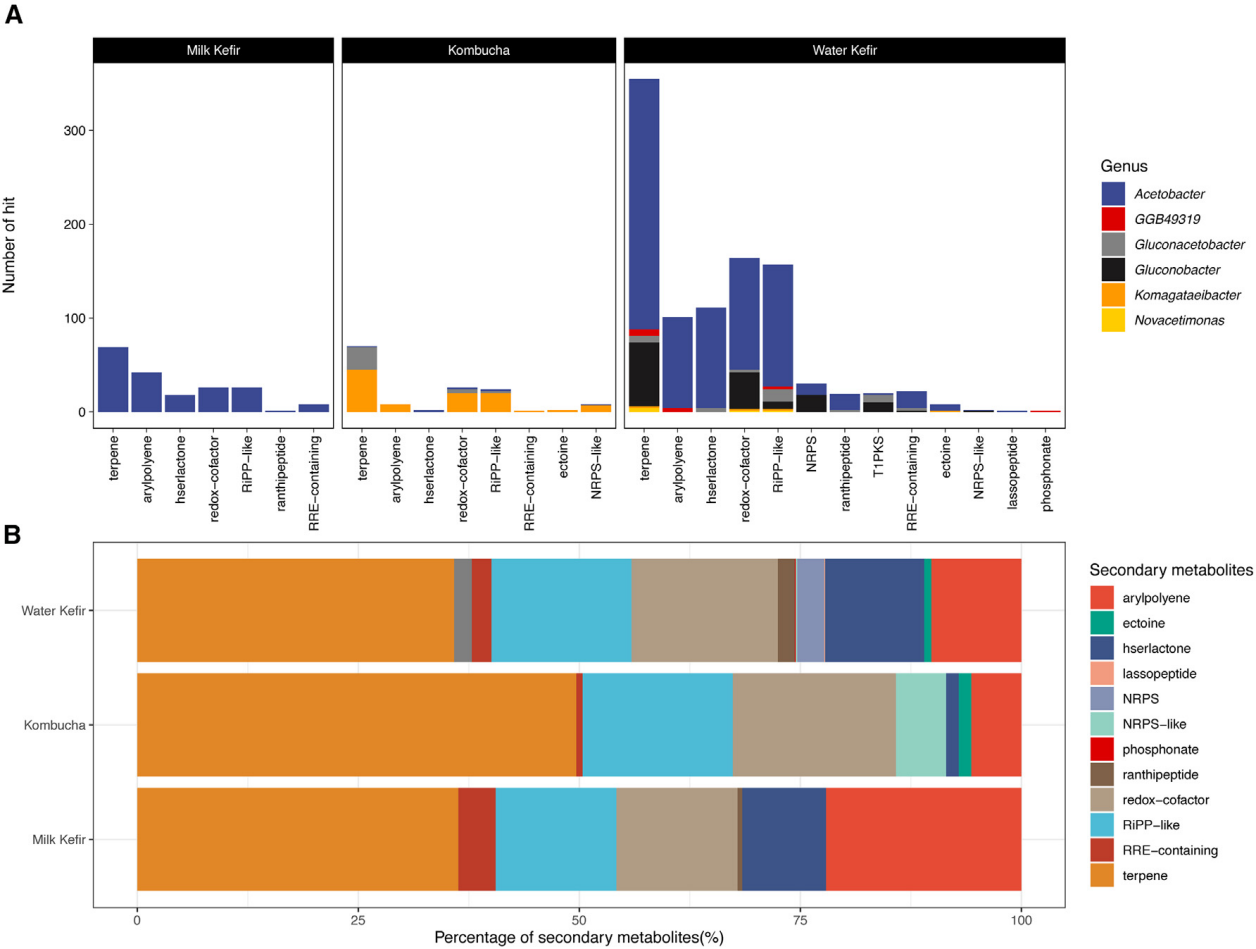
Secondary metabolite cluster annotation of AABs MAGs derived from kombucha, water kefir, and milk kefir (A) The diversity and composition of secondary metabolite cluster identified in AABs MAGs. (B) The relative abundance of each secondary metabolite cluster detected in kombucha, water kefir, and milk kefir.

The set of genes involved in bacterial secondary metabolism are of great interest being responsible for the production of bioactive compounds, some of which are of potential pharmaceutical value, including antibiotics, cholesterol-lowering drugs, and antitumor drugs.^26^ In this study, we used antiSMASH to predict secondary metabolic pathways encoded by the AABs MAGs from milk kefir, water kefir, and kombucha. antiSMASH uses a rule-based cluster detection approach and can identify 71 different types of secondary metabolite biosynthetic gene clusters.^27^ The results revealed a total of 1,322 hits across 12 categories of secondary metabolites. The three most commonly detected categories of secondary metabolite-associated genes in milk kefir, water kefir, and kombucha-associated AABs are those associated with terpenes, redox-cofactors, and aryl polyenes (Figure 4A). Further inspection showed that the percentage of aryl polyene-associated genes in AABs from milk kefir is significantly (*p* < 0.05) higher than those in the AABs from kombucha or milk kefir (Figure 4B), while the percentage of terpene-associated genes was higher in kombucha-associated AABs (Figure 4B).

### Analysis of AMR genes revealed the differences of antibiotic resistance potential in AABs MAGs across different sample types

**Figure 5:**
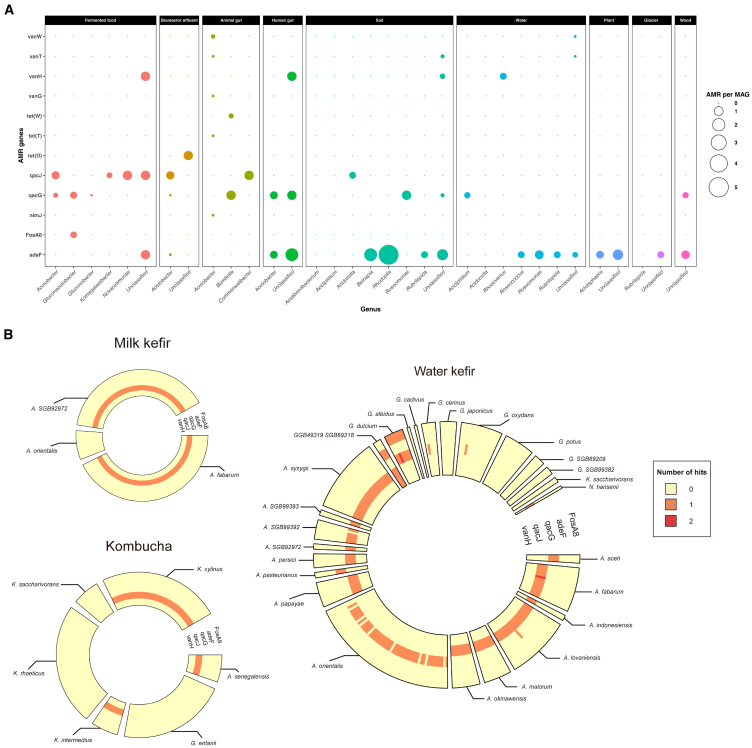
The diversity and composition of AMR genes in AABs MAGs (A) The AMR genes detected in 609 AABs MAGs derived from different sample origin (337 high-quality AABs MAGs referred to cFMD and 272 AABs MAGs downloaded from NCBI). (B) The distribution of AMR genes in AABs MAGs across the three types of fermented foods.

Among the whole set of 609 AABs MAGs, we detected 485 antimicrobial resistance (AMR) genes. The types of AMR genes present in AABs MAGs varied with their metagenome sample types (Figure 5A). Of the AMR genes detected, *qacJ* and *qacG* (associated with resistance to disinfecting agents and antiseptics) were more prevalent in AABs MAGs that were generated from food-related metagenomic samples (Figure 5A). However, among environment samples (soil, water, plant, etc.), less *qacJ* and *qacG* were found, while *adeF* (associated with resistance to the antibiotic tetracycline) being more abundant (Figure 5A).

A more specific examination of AMR genes associated with the MAGs from kombucha, water kefir, and milk kefir was performed. According to Comprehensive Antibiotic Resistance Database, 249 putative AMR genes were identified across the 313 AABs MAGs. Those AMR genes can be assigned to three general resistance mechanisms, i.e., antibiotic target alteration, antibiotic inactivation, and antibiotic efflux. There is at least one putative AMR gene per MAG in most AABs species (Figure 5B). In milk kefir and kombucha AABs MAGs, all AMR genes detected resembled *qacJ*. A greater variety of putative AMR genes were found in water kefir. These included *adeF* (resistance to tetracycline antibiotic), *fosA8* (resistance to fosfomycin), and *qacG* and *vanH* (resistance to vancomycin) (Figure 5B). Those of greater concern were specifically associated with *GGB49319* (the unknown genus-level genome bin; *adeF* and *vanH*) and *Gluconacetobacter dulcium* (*fosA8*) (Figure 5B). As shown in Figure 5B, the distribution of AMR genes in most AABs species is homogeneous. However, exceptions existed in that, for example, *qacG* was found in some, but not all, *Acetobacter orientalis* MAGs from water kefir.

### Examination of metabolic capabilities reveals functional potentials across the AABs species found in fermented foods

**Figure 6:**
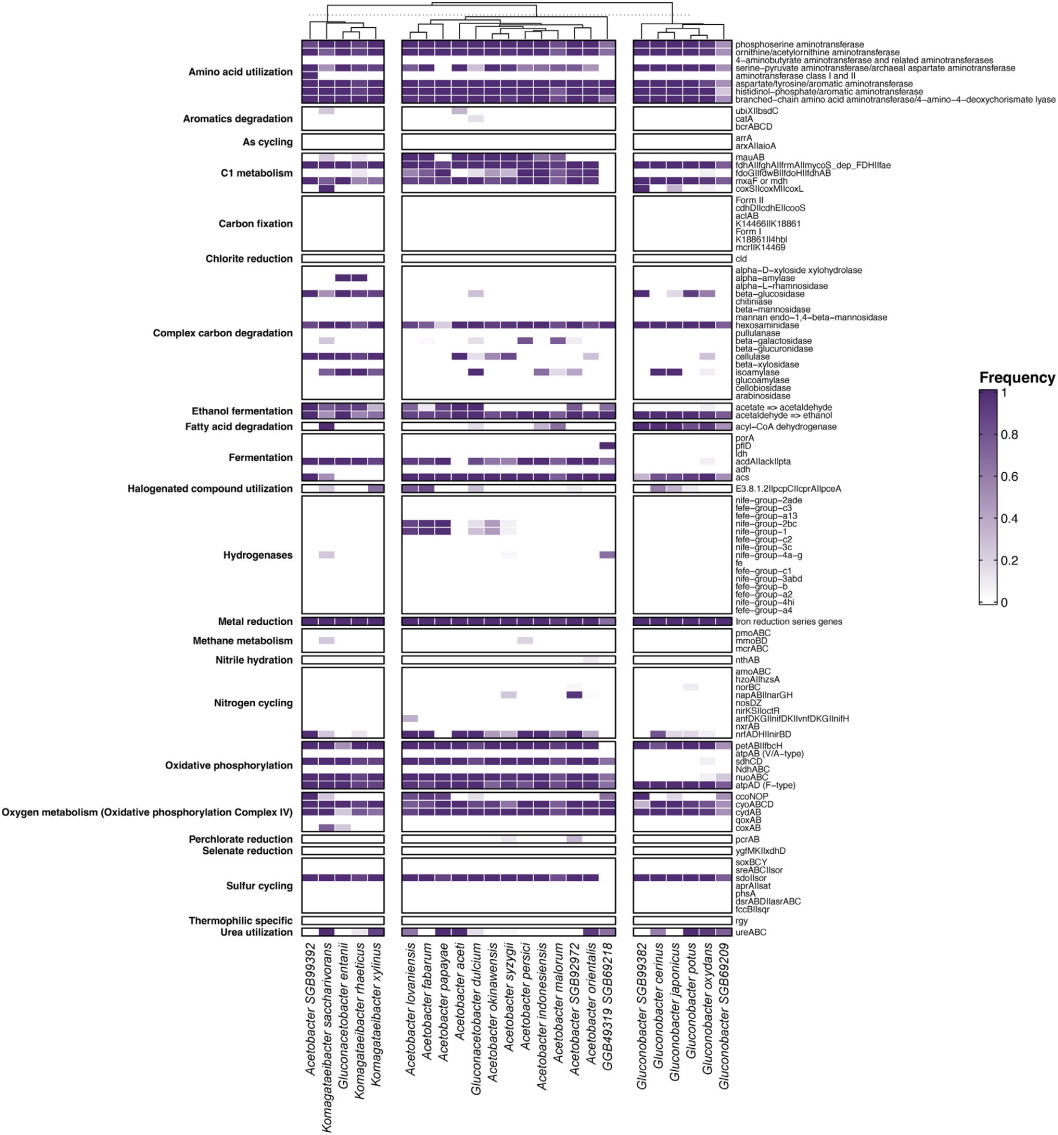
Metabolic potential of all 337 high-quality fermented food AABs MAGs In the cluster heatmap, white squares indicate absence of a given gene in all MAGs, and the darkest purple indicates presence in all of them.

In order to further explore other functional traits of AAB, we used METABOLIC to predict the metabolic profiles of input MAGs and elucidate if the presence-absence patterns of these pathways can distinguish AABs species. METABOLIC has several genome-scale workflows that relate to the annotation of microbial genomes, motif validation of biochemically validated conserved protein residues, metabolic pathway analyses, and calculation of contributions to individual biogeochemical transformations and cycles.^28^ Investigating the metabolic potential across the different AABs species highlighted three major clusters, which predominantly contained *Gluconobacter* spp., *Acetobacter* spp., and *Komagataeibacter* spp., respectively (Figure 6). We observed the putative fermentation genes for acetogenesis (*acdA*, *ack*, and *pta*) to be widespread across *Acetobacter*, *Komagataeibacter*, and *Gluconacetobacter* species (Figure 6). However, these genes were not identified in *Gluconobacter* spp. This is consistent with the ability of *Acetobacter* to oxidize acetate and lactate, a trait that is absent among *Gluconobacter*. We also found that the *acs* gene, encoding the enzyme responsible for the conversion of acetate to acetyl-CoA, is widely distributed in *Acetobacter* spp. and *Gluconobacter* spp., while only a few hits were found in *Komagataeibacter* spp (Figure 6). Genes predicted to encode cellulases were found in kombucha-associated AABs MAGs, such as *Komagataeibacter* spp. and *Gluconacetobacter* spp (Figure 6). This feature is likely selected for by the high cellulose content that can be an abundant carbon source for the growth of AABs in kombucha. As for hydrogenases, only NiFe hydrogenase genes were detected in some *Acetobacter* spp (*A. lovaniensis*, *A. fabarum*, *A. papaya*, etc.).

**Figure 7:**
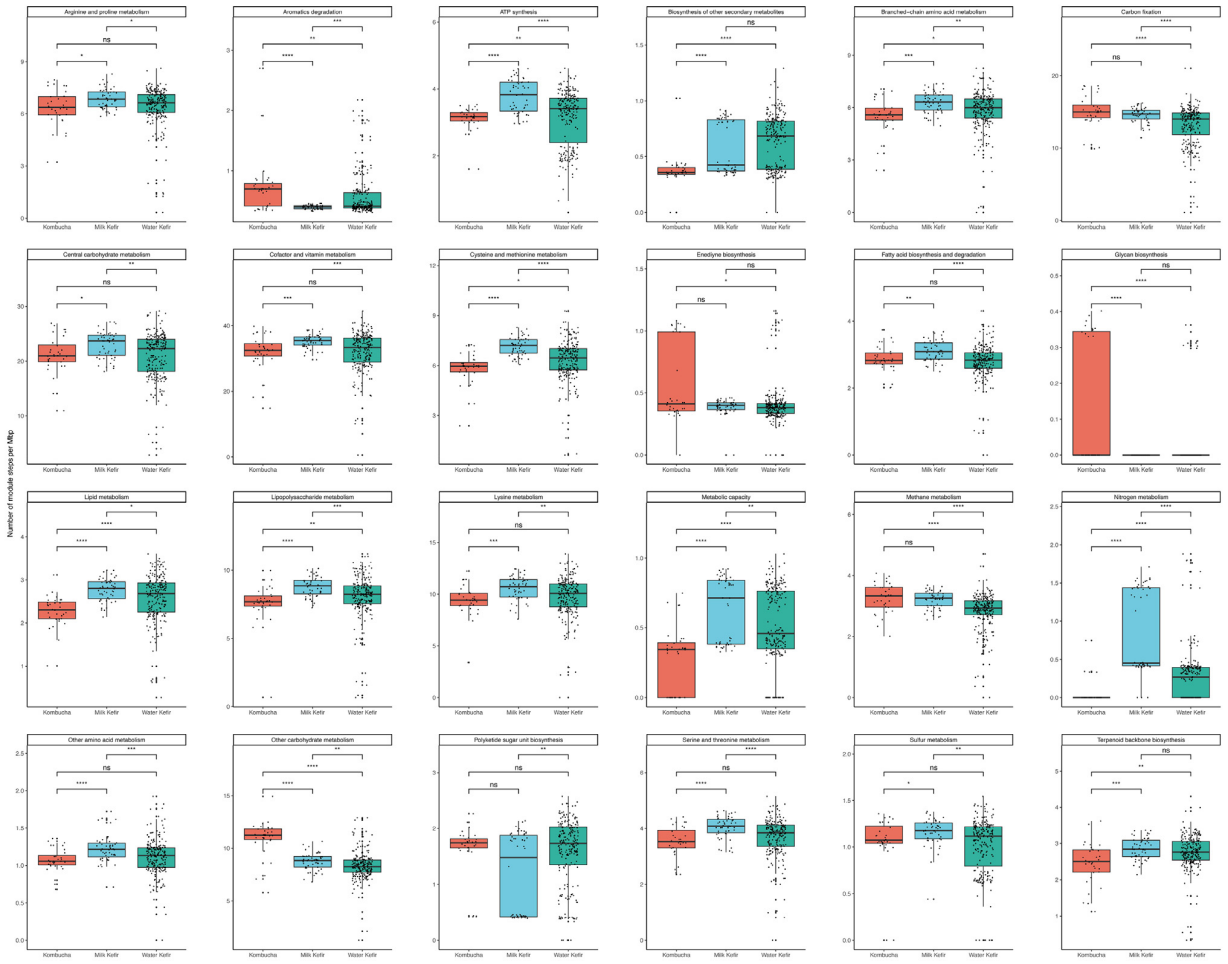
Comparison of the number of KEGG module steps per Mbp across kombucha, water kefir, and milk kefir Only KEGG categories, which have a significant difference, were shown in this figure. Stars in boxplots indicate significant differences (Wilcoxon non-parametric test, ∗*p* < 0.05, ∗∗*p* < 0.01, ∗∗∗*p* < 0.001, ∗∗∗∗*p* < 0.0001 and ns: not significant). Data are represented as mean ± SEM.

We also calculated the number of module steps per Mbp in AABs MAGs from milk kefir, water kefir, and kombucha to analyze the difference of functional categories of AABs community in different fermented foods. Functional categories include different but related metabolic pathways (Kyoto Encyclopedia of Genes and Genomes [KEGG] modules), and each module comprises multiple specific metabolic reactions (module steps).^28^ This provided a number of notable findings. Firstly, the AABs in milk kefir contained a greater number of module steps relating to amino acid-related metabolic reactions (i.e., metabolism of amino acids), per Mbp (Figure 7). Similarly, the module steps relating to fatty acid-related metabolism (lipid metabolism, fatty acid biosynthesis and degradation, lipopolysaccharide metabolism, etc.) per Mbp in milk kefir AABs were also significantly higher than the other two fermented foods. However, in the case of nitrogen metabolism, kombucha AABs had a significantly higher number of module steps than the other two fermented foods, while the reverse was observed with respect to glycan biosynthesis modules (Figure 7).

### The presence of PHAGCs differs between *Gluconobacter* spp. and other AABs species

An investigation of the dispersion of putative health-related gene clusters (PHAGCs) in these fermented food AABs MAGs provided insights into the probiotic potential of AAB. PHAGCs were divided into three broad categories, namely “survival,” “colonization,” and “modulation.” Survival genes were reported to be important for surviving the low pH of the stomach or the bile salts of the small intestine.^29^ Also, genes which were believed to be important in colonizing the gut microbiome were labeled as colonization. Besides, modulation genes were shown to have the ability to affect the host phenotype in other ways, such as stimulating the host immune system through the production of D-phenyl-lactic acid^30^ or γ-aminobutyric acid.^31^

**Figure 8:**
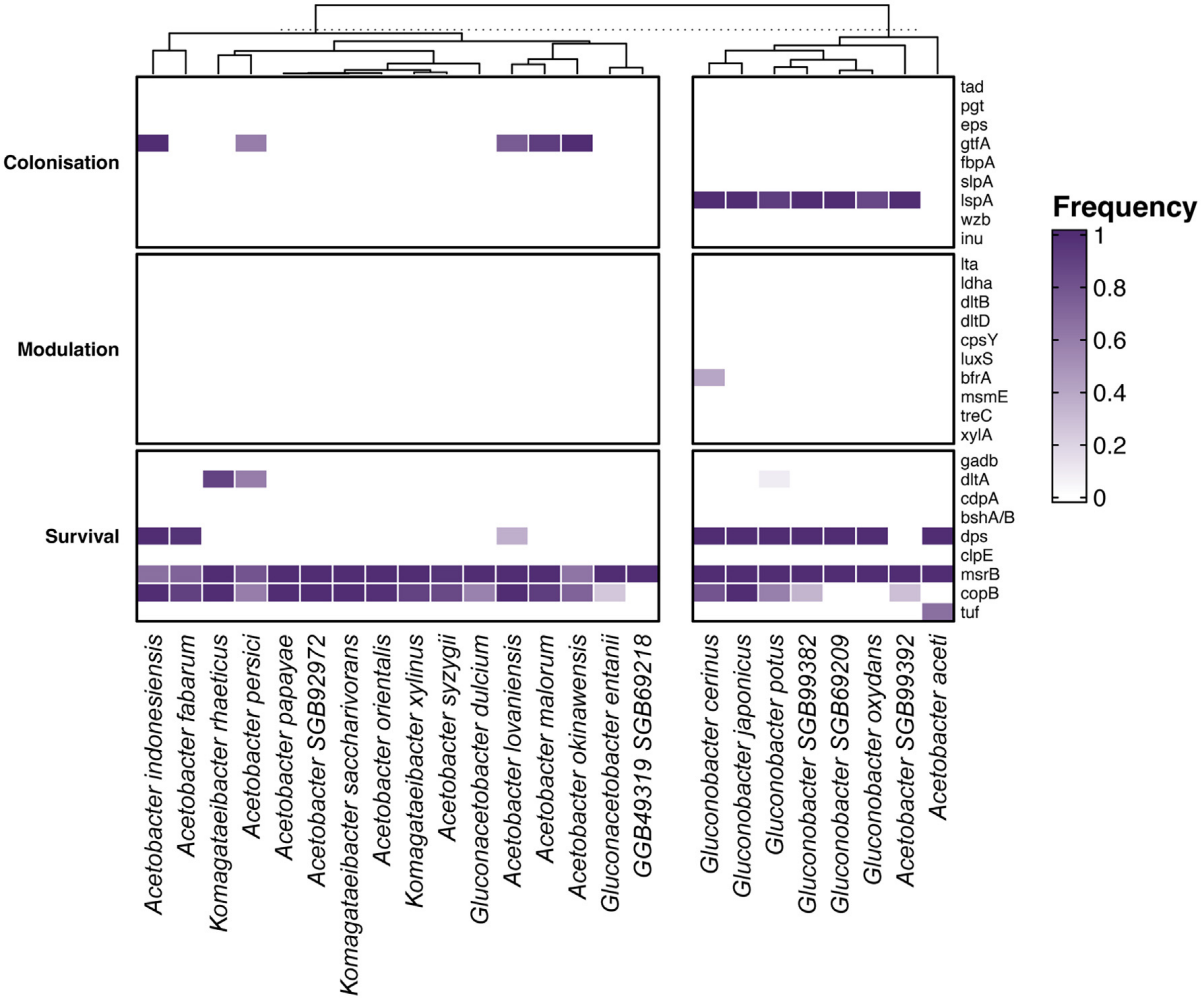
PHAGCs screening across all 337 high-quality fermented food AABs MAGs In the cluster heatmap, white squares indicate the absence of a given gene in all MAGs, and the darkest purple indicates presence in all of them.

Generally, the hits of PHAGCs in AABs MAGs were mainly binned in the colonization and survival categories (Figure 8). As depicted in the heatmap generated, *msrB* and *copB* (encoding a peptide methionine sulfoxide reductase and copper-exporting P-type ATPase B, respectively) were the 2 main PHAGCs detected in AABs MAGs (Figure 8). It was also evident that *Gluconobacter* spp. had a different PHAGC profile than other AAB, with a high prevalence of *lspA* and *dps* (encoding lipoprotein signal peptidase and DNA protection during starvation protein, respectively) that were almost absent elsewhere.

## Discussion

The group of gram-negative bacteria that are capable of oxidizing ethanol to acetic acid even under highly acidic conditions is known as AAB.^32^ This special ability of AABs is the basis for their key role in the vinegar fermentation process. AABs can also be used for the generation of other metabolic products, such as gluconic acid, l-sorbose, and bacterial cellulose.^33^ Another important characteristic of AABs is that most substrates absorbed by AABs are efficiently converted into products because of the outstanding catalytic abilities of dehydrogenases.^34^ Thus, those features mean that AABs are very suitable for application in biotechnology. Despite their peculiar features, AABs are much less extensively studied than the LAB, the other major category of fermented food-associated bacteria. A greater understanding of the characteristics and metabolic potential of AABs has the potential to promote processes optimization and uncover other previously unknown significant applications that can be performed by these bacteria, especially when it comes to fermented foods and beverages.

The first step in this process is a requirement to accurately identify the AABs present in fermented foods. It should be noted that AABs taxonomy has been undergoing extension revision,^35^ and the provision of additional data can provide even greater clarity. In this study, we examined 337 high-quality AABs MAGs derived from a variety of fermented foods and integrated with 272 public AABs MAGs from various environments, which could greatly contribute to the more accurate and rapid identification of fermented food AAB. AABs often occur as viable but not culturable cells during spontaneous food fermentation processes, which makes it cumbersome to isolate or cultivate AAB.^36^ Indeed, this is thought to be the reason why AABs are only sporadically recovered throughout the fermentation and maturation process,^37^^,^^38^ although they are believed to play an important role in several processes. For instance, according to previous research, AABs are not always detectable in water kefir, differing even between studies from the same group.^39^^,^^40^ In this study, culture-independent approaches have revealed 26 AABs species in water kefir, many of which have not been reported before. The interactions occurring between those undiscovered, uncultivable species and important compounds need to be explored. Overall, we identified 9 putative novel species across the fermented food samples studied, two of the most noteworthy being *SGB92972* and *GGB49319 SGB69218*. *SGB92972* was widely found across cFMD samples, including water kefir, milk kefir, and sourdough. According to the FastANI results, the closest genome in the NCBI database for *SGB92972* is *Acetobacter cibinongensis* (91.42% identity). Notably, *A. cibinongensis* have been mainly found in tropical fruits and flowers, which can be raw materials for some kinds of water kefir, but its presence in milk kefir and sourdough is unusual. *GGB49319 SGB69218* was identified in three water kefir samples, and the closest previously sequenced relative is *Acidisphaera rubrifaciens* (78.05% identity), a species found in acidic hot springs and mine drainage systems.^41^ The detection of these putatively novel species was also noted in a parallel study, relating to water kefir metagenomes, from our lab (manuscript submitted). The combined phylogenetic analysis with a large collection of MAGs from the NCBI database also provided deeper insights. Although phylogenetically diverse, closely related MAGs generally clustered in a manner that reflected their origin. However, an exception was observed with respect to several MAGs generated from gut samples that were found to be phylogenetically close to some MAGs recovered from fermented food samples. This is notable as, unlike the case for the LAB, there is only a limited literature relating to the interaction between AABs and the gut microbiome and/or their potential to impact health. Further investigation of those overlap species will be important with a view to identifying potential links between gut and food AAB.

Although AABs are commonly found in fermented foods and beverages, their role often remains undetermined. Thus, while determining that taxonomy is important, gaining a greater understanding of the metabolic potential of these AABs is also critical. As key growth substrates, carbohydrates play a key role in shaping the microbiota of fermented foods, especially the ability to degrade carbohydrates provides an advantage for the growth of certain bacteria.^42^ Our CAZyme profiling analysis of all high-quality MAGs showed that GH- and GT-encoding genes were the two most prevalent CAZyme families among fermented food-associated AAB. The products of GH family genes mainly hydrolyze glycosidic linkages, while GT family proteins are known to catalyze the formation of glycosidic linkages using activated donors to transfer sugars to specific acceptors.^25^ In line with our study, Li et al. have reported an abundance of GH (30%) and GT (45%) modules among the CAZyme-encoding genes in sourdough started by AAB.^43^ Our pairwise comparison of CAZyme genes across fermented foods also revealed that there are significantly more genes coding for CAZymes containing CBM48 in kombucha than milk kefir and water kefir. Notably, it has been suggested that the abundance of CBM48 in gut microbiota is correlated with the expression of AMP-activated protein kinase β (AMPK β, an important target for AMPK activator) in the intestinal epithelial,^44^ revealing a potential route via which consumption of AABs in kombucha could impact health. In addition, we established that there are significant differences in the types of GH and GT genes among the AABs found in milk kefir and water kefir. This is likely driven by the differences in the diversity of carbohydrates present in these two fermented foods. Due to the addition of fruit components during the fermentation process, water kefir can contain a more diverse range of carbohydrates, including polysaccharides, e.g., cellulose; oligosaccharides, e.g., maltose and sucrose; and some monosaccharides, e.g., glucose and fructose. In contrast, the carbohydrates in milk kefir mainly consist of lactose and galactose. Another finding is that GT2 and GT4 are significantly more prevalent in water kefir and kombucha AABs MAGs than milk kefir AABs MAGs. Those two subfamilies of CAZyme have a wide range of activities including but not limited to the degradation of oligo- and polysaccharides.^30^ Thus, the reason why more GT2 and GT4 genes were detected in kombucha and water kefir may be the high polysaccharide content that provides an abundant carbon source for the growth of microorganisms in fermented products.^45^ GH3 and GH5 have also been found to be present in significantly greater abundance among kombucha-associated AABs than for AABs in other fermented foods. GH3 and GH5 are mainly responsible for hydrolyzing cellulose and hemicellulose, respectively. Notably, kombucha is traditionally produced by fermenting a symbiotic culture of bacteria and yeast, around which a cellulose film is formed. The breakdown of cellulose and hemicellulose produces monomeric sugars, which diminish astringency and bitterness while enhancing the sweetness and smooth flavor of the tea liquor. It may thus be that GHs produced by *Komagataeibacter* spp. and *Gluconacetobacter* spp., which comprise a major part of the kombucha AABs community, can have a major impact on the attributes of different kombuchas.

Although the isolation of antibiotic-resistant bacteria from fermented foods is not unusual,^46^ it is important to understand the distribution of AMR genes among such microbes. From our analysis, a total of 249 AMR genes were identified in 262 AABs MAGs, which were distributed across 24 AABs species. The most prevalent AMR genes in our dataset are *qacJ* and *qacG*, which encode proton-motive force-dependent export pumps conferring resistance to a broad range of structurally diverse hydrophobic compounds.^47^ A plasmid-borne *qacJ* determinant has been found in several *Staphylococcus* spp. and has the potential to be spread by horizontal gene transfer.^48^ In this study, *qacJ* was found to be widely distributed across 16 species across water kefir, milk kefir, and kombucha. It should be noted that two species, *GGB49319 SGB69218* and *G. dulcium*, both have more than one resistance gene per MAG, which is higher than other AABs species. These bacteria may act as a reservoir of antibiotic-resistant genes,^49^ but further investigation is required. It was notable that the presence of *qacG* in *Acetobacter orientalis* was associated only with MAGs derived from water kefir, possibly reflecting the different subspecies they represent.

The results from METABOLIC also provided a series of interesting insights. Notably, with respect to the ethanol fermentation process, all *Acetobacter* spp. were predicted to have the ability to consume ethanol, while only *Komagataeibacter* spp. and *Gluconacetobacter* spp. can complete the process of converting acetate to ethanol. The former is consistent with the findings of Abigail et al. who demonstrated that ethanol in water kefir can be entirely consumed and oxidized to acetic acid by the widespread dissimilatory route of *Acetobacter* spp*.*^50^ Apart from this, according to our results (Figure 6), the putative novel species *Acetobacter SGB99392* has very similar metabolic features to *Komagataeibacter* spp. and *Gluconacetobacter* spp., thereby making its taxonomic assignment complicated. The future isolation and characterization of strains of these microorganisms should provide clarity. Additionally, no FeFe hydrogenases were detected in all AABs MAGs, while several NiFe hydrogenases were identified in some *Acetobacter* spp (*A. lovaniensis*, *A. fabarum*, *A. papaya*, etc.). NiFe group 1 hydrogenases could be playing a vital role in nitrate (NO_3_^−^), sulfate (SO_4_^2−^), and iron (Fe^3+^) reduction: these molecules can act as acceptors of electrons coupled to H_2_ oxidation in anoxic conditions,^51^ which suggests that those organisms may stay energized by switching from hydrogenotrophic aerobic respiration when oxygenated to hydrogenogenic fermentation during anoxia.^52^ We also found that the AABs in milk kefir contain a greater number of amino acid and fatty acid-related KEGG module steps per Mbp, most likely reflecting their adaptation to dairy environments. According to previous research, aromatic amino acids (tyrosine, phenylalanine, and tryptophan) and amino acids containing sulfur (methionine, cysteine, and taurine) exhibit strong antioxidant activity by scavenging-free radicals or reactive oxygen species.^53^ Hence, AABs in milk kefir may contribute to the antioxidant capacity of the beverage.

Analysis of PHAGCs highlighted that colonization and survival genes were more common in MAGs from fermented food AAB. More specifically, the genes coding for peptide methionine sulfoxide reductase and copper-exporting P-type ATPase B (*msrB* and *copB*) were present in most AABs species. The peptide methionine sulfoxide reductase can catalyze the reduction of methionine sulfoxide in proteins back to methionine, and growing evidence showed that this enzyme plays an important role in protecting cells against oxidative damage.^54^ Apart from this, the product of stress-related genes involved in copper detoxification (*copB*) can act as an exporter, thereby preventing the accumulation of copper in the cytoplasm.^55^ The efficient transport of toxic compounds is vital for the persistence of bacteria in the gastrointestinal tract.^55^ Another interesting finding is that the presence of some PHAGCs (*lspA* and *dps*) differed significantly between *Gluconobacter* spp. and other AABs species. The product of *lspA* was reported to have a significant contribution to epithelial cell adhesion by bacteria.^56^ The difference in *dps* gene distribution is notable as these genes have been shown to be involved in several types of stress adaptation in *Escherichia coli*, including adaptation to oxidative and pH stress, irradiation, and metal toxicity.^57^^,^^58^ Although *in vivo* studies are required to directly examine probiotic potential, these results still have provided some insights in this regard.

It should be noted that this study relied exclusively on the analysis of assembled data, i.e., MAGs, rather than unassembled date to determine functional potential. Although this represents a limitation, as genes from less abundant AABs may be overlooked, this approach was necessary to ensure that only AAB-associated genes were considered.

In conclusion, in this research article, we provide a comprehensive and high-resolution analysis to show taxonomy, distribution, and predicted function of AABs in fermented food based on a large dataset of high-quality AABs MAGs. Specifically, we investigate (1) how AABs species were distributed in fermented foods and their phylogenetic relations with MAGs reconstructed from different environments, (2) what valuable secondary metabolites or persistence of reservoirs of antimicrobial resistance could be produced by fermented food AAB, and (3) which metabolic pathways and PHAGCs contrast between different AABs species or different fermented food AABs community. Additionally, we also found several putative novel species and a putatively novel genus, which may be interesting to be further studied. Overall, our study illustrates the importance of analysis based on MAG datasets, which helps expand our understanding of fermented food AABs communities and provides a better understanding of the fermentation process. This knowledge can potentially contribute to the production of higher quality products as the AABs community can affect the production of metabolites such as organic acids, which are associated with potential health benefits, as well as sensory properties.

### Limitations of the study

In this study, the annotation and validation of MAGs rely heavily on existing databases, which may not be comprehensive, leading to gaps in knowledge. As new genomes are sequenced and added to databases, the classification and annotation of previously analyzed MAGs may change, requiring re-analysis to maintain consistency with the latest data.

## Resource availability

### Lead contact

Further information and requests for resources and reagents should be directed to and will be fulfilled by the lead contact, Paul D. Cotter (paul.cotter@teagasc.ie).

### Materials availability

This study did not generate new reagents.

### Data and code availability

• The table for the accession number of sequence data for cFMD can be found at https://github.com/SegataLab/cFMD.
• Computer code used in data analysis is available from the corresponding author upon reasonable request.
• Any additional information required to reanalyze the data reported in this paper is available from the lead contact upon request.

## Acknowledgments

We would like to acknowledge the feedback of our colleagues in the Cotter laboratory at Teagasc research center, with whom we discussed this work, especially Dr. John Leech. This research was funded by the 10.13039/100010661H2020-funded MASTER project and through the EU’s 10.13039/100019637Horizon Europe DOMINO project (grant number 101060218), by 10.13039/501100001602Science Foundation Ireland (SFI) under grant number SFI/12/RC/2273_P2 (APC Microbiome Ireland), by SFI together with the 10.13039/501100001584Irish Department of Agriculture, Food and the Marine under grant number SFI/16/RC/3835 (VistaMilk), and by 10.13039/501100001588Enterprise Ireland and industry in the Food for Health Ireland (FHI)-3 project, under grant number TC/2018/0025. The MASTER EU Consortium has received funding from the European Union’s 10.13039/501100007601Horizon 2020 research and innovation program under grant agreement no. 818368.

## Author contributions

E.Z. and P.D.C. conceived the study idea and design. E.Z. conducted bioinformatics analysis. E.Z. wrote the manuscript with contributions from M.J.C., S.B., N.S., N.C., and P.D.C. P.D.C. supervised the project.

## Declaration of interests

The authors declare no competing interests.

## STAR★Methods

### Key resources table

**Table:** 

REAGENT or RESOURCE	SOURCE	IDENTIFIER
**Deposited data**		

curatedFoodMetagenomicData (cFMD)	https://github.com/SegataLab/cFMD	

**Software and algorithms**		

CheckM	Parks et al.^59^	https://github.com/Ecogenomics/CheckM
dRep	Olm et al.^60^	https://github.com/MrOlm/drep
GTDB-Tk	Chaumeil et al.^61^	https://github.com/Ecogenomics/GTDBTk
iTOL	Letunic et al.^62^	https://itol.embl.de
Prodigal	Hyatt et al.^63^	https://github.com/hyattpd/Prodigal
antiSMASH	Blin et al.^27^	https://github.com/antismash
dbCAN3	Zheng et al.^64^	https://github.com/linnabrown/run_dbcan
METABOLIC	Zhou et al.^28^	https://github.com/AnantharamanLab/METABOLIC
R version 4.2.2	R Core Team	https://www.r-project.org/

### Method details

#### Acetic acid bacteria metagenome-assembled genomes collection

In order to collect the reconstructed genomes from food metagenomes and belonging to AAB, we queried the curatedFoodMetagenomicData (cFMD, https://github.com/SegataLab/cFMD, https://zenodo.org/records/10891047) for MAGs belonging to *Acetobacteraceae* family. We further filtered the 523 downloaded MAGs according to CheckM^59^ (ver. 1.0.18, option ‘lineage_wf’) parameters, keeping only those with high quality (completeness > 90% and contamination < 5%). Finally, the 343 HQ MAGs were dereplicated using fastANI algorithm in dRep^60^ (ver. 3.2.0) with a threshold of 99% average nucleotide identity (ANI), which led us to the final set of 337 MAGs belonging to AABs and reconstructed from food samples. Contextually, we retrieved the relative metadata reporting the published study (dataset), the food categorization of the original sample and the taxonomic label assigned to the MAG. The taxonomy followed the Species-level Genome Bins (SGBs) architecture,^65^ where clusters of both isolated species and MAGs are defined based solely on genomic distances and can be therefore assigned as kSGB (known SGB, i.e. containing at least one isolate genome) or uSGB (unknown/unknownfood SGB, uSGBs/ufSGB). The latter, containing only MAGs, represent metagenomically reconstructed yet-to-be-characterized species.

In addition, 272 MAGs were downloaded from the NCBI database to enhance the diversity of the dataset and contribute to a comparative genomic analysis (Table S1). This selection was created by the investigation of all high quality (excluding “anomalous” filter and ‘representative’ RefSeq filter) and taxonomically accurate (taxonomy “OK” filter) *Acetobacteraceae* MAGs available at the NCBI assembly database. Only bacterial MAGs for which there was robust documentation with respect to their source and originated from human, animal, food or environmental samples (e.g. from water, soil and glaciers) were included.

#### Phylogenetic analysis

The phylogenetic tree was built based on the sequences of these 609 MAGs by using GTDB-Tk^61^ (ver. 2.2.5) with default parameters. To be specific, the aligned protein sequences were produced by using the ‘classify_wf’ workflow in GTDK-Tk and the use of the ‘infer’ workflow to infer the phylogenetic tree (parameters: default). The phylogenetic trees were visualized using iTOL^62^ (ver. 6.8.1) and combined with other metadata, including the taxonomy information, the quality of MAGs and length of genomes.

#### Functional annotation

Proteins of the contigs were annotated using Prodigal^63^ (ver. 2.6.3) with parameters “-p meta”. Secondary metabolites clusters were annotated using antiSMASH^27^ (ver. 6.1.1) with parameters “—genefinding-tool prodigal”. The CAZymes genes were annotated by using run_dbcan4^64^ (ver. 4.0.0), and considered to be positive hits only if the genes were found by both HMM and DIAMOND based approaches. Antimicrobial resistance (AMR) genes were annotated using RGI (ver. 6.0.1) (default parameters, “-t contig –local –clean”) based on the Comprehensive Antibiotic Resistance Database^66^ (CARD). To analyze the metabolic potential of AABs in different fermented foods, the protein translation files of all high-quality MAGs in this study were processed using METABOLIC^28^ (ver. 4.0), using the METABOLIC-G workflows with default parameters.

#### Putative health related genes cluster screening

PHAGC screening was performed according to Leech et al.^19^ More specifically, all 337 high quality MAGs were annotated by using Prokka (ver. 1.14.6) (default parameters). The presence or absence of putative health related gene clusters (PHAGCs) was determined by the Prokka output. The list of names and products of PHAGCs can be found in Table S2. PHAGCs were classified into 3 different categories (modulation, survival and colonization) based on their function.

### Quantification and statistical analysis

Statistical analysis in this study was performed in R (ver. 4.2.2) (https://www.r-project.org/). The ggplot2 package^67^ (ver. 3.4.2) and ggalluvial package (ver. 0.12.4) was used to visualize data and ComplexHeatmap^68^ (ver. 2.14.0) was employed to construct the cluster heatmap. The Wilcoxon non-parametric test was used to determine if significant differences existed between pairs of groups; P-values <0.05 were considered statistically significant.

## Supplemental information

Table S1. Metadata for the sequencing data or MAGs used in this study, related to STAR Methods Sheet 1. Metadata for cFMD metagenomic sequencing data. Sheet 2. Metadata for high-quality AAB MAGs used in this study. Sheet 3. Metadata for AAB MAGs downloaded from NCBI that used in this study.

Table S2. The list of putative health-related genes cluster, related to Figure 8
